# Regenerative and fibrotic pathways in canine hepatic portosystemic shunt and portal vein hypoplasia, new models for clinical hepatocyte growth factor treatment

**DOI:** 10.1186/1476-5926-4-7

**Published:** 2005-12-07

**Authors:** Bart Spee, Louis C Penning, Ted SGAM van den Ingh, Brigitte Arends, Jooske IJzer, Frederik J van Sluijs, Jan Rothuizen

**Affiliations:** 1 Department of Clinical Sciences of Companion Animals, Faculty of Veterinary Medicine, Utrecht University, Utrecht, The Netherlands; 2 Department of Pathobiology, Faculty of Veterinary Medicine, Utrecht University, Utrecht, The Netherlands

## Abstract

**Background:**

We analyzed two spontaneous dog diseases characterized by subnormal portal perfusion and reduced liver growth: (i) congenital portosystemic shunts (CPSS) without fibrosis and (ii) primary portal vein hypoplasia (PPVH), a disease associated with fibrosis. These pathologies, that lack inflammation or cholestasis, may represent simplified models to study liver growth and fibrosis. To investigate the possible use of those models for hepatocyte growth factor (HGF) treatment, we studied the functionality of HGF signaling in CPSS and PPVH dogs and compared this to aged-matched healthy controls.

**Results:**

We used quantitative real-time polymerase chain reaction (Q-PCR) to analyze the mRNA expression of HGF, transforming growth factor β1 (TGF-β1), and relevant mediators in liver biopsies from cases with CPSS or PPVH, in comparison with healthy control dogs. CPSS and PPVH were associated with a decrease in mRNA expression of HGF and of MET proto-oncogene (c-MET). Western blot analysis confirmed the Q-PCR results and showed that intracellular signaling components (protein kinase B/Akt, ERK1/2, and STAT3) were functional. The TGF-β1 mRNA levels were unchanged in CPSS whereas there was a 2-fold increase in PPVH indicating an active TGF-β1 pathway, consistent with the observation of fibrosis seen in PPVH. Western blots on TGF-β1 and phosphorylated Smad2 confirmed an activated pro-fibrotic pathway in PPVH. Furthermore, Q-PCR showed an increase in the amount of collagen I present in PPVH compared to CPSS and control, which was confirmed by Western blot analysis.

**Conclusion:**

The pathophysiological differences between CPSS and PPVH can adequately be explained by the Q-PCR measurements and Western blots. Although c-MET levels were reduced, downstream signaling seemed to be functional and provides a rational for HGF-supplementation in controlled studies with CPSS and PPVH. Furthermore both diseases may serve as simplified models for comparison with more complex chronic inflammatory diseases and cirrhosis.

## Background

Chronic liver disease is characterized by decreased regeneration of hepatocytes and increased formation of fibrous tissue. These characteristics may be the sequel of various chronic processes such as cholestasis, viral infections, toxin exposure, and metabolic disorders. Dogs have complex liver diseases such as hepatitis and cirrhosis which are highly comparable with the human counterparts. Moreover, coding sequences of dogs proved highly homologous to the human sequences [1], especially compared to the rodent genome. Thus, dogs may fulfill a role as a spontaneous animal model in between toxin-induced or surgical models in rodents, and spontaneous diseases in man. The complex interplay of many factors active in chronic liver disease makes it difficult to unravel the roles of different individual pathogenetic pathways. Dogs display liver diseases, which are potentially valuable models to compare complex with simple pathologic entities.

We have chosen these two congenital dog diseases for comparative analysis of liver growth/regeneration, fibrosis, and hepatic homeostasis: congenital portosystemic shunt (CPSS) and primary portal vein hypoplasia (PPVH). CPSS is characterized by an abnormal single large communication between the portal vein and a major systemic vein (cava or azygos). This results in the virtual absence of portal vein perfusion to the liver from birth onwards. Liver growth remains nearly absent but there is essentially no liver pathology [2,3]. PPVH is a developmental abnormality in which the terminal vein branches are not or only partially present and, in most cases, in combination with congenital portal fibrosis, but without inflammation [4]. PPVH is associated with portal hypertension and reduced liver growth. Thus, these two congenital diseases represent relatively simple models for reduced liver growth associated with fibrosis (PPVH) or without fibrosis (CPSS). Both diseases have a decrease in liver growth due to differences in portal perfusion which results in a massive reduction of liver size.

Because hepatocyte growth factor (HGF) is one of the most important genes involved in liver growth/regeneration [5-7], abnormal expression of HGF could play a major role in the decreased liver size in CPSS or PPVH. Therefore, treatment of dogs with HGF could be a possible therapeutic approach. A pre-requisite for treatment is that HGF signaling components are unaffected in those dogs. Consequently, we focused on measuring gene products involved in signaling of HGF and counteracting transforming growth factor β1 (TGF-β1). All biological responses induced by HGF are elicited by binding to its receptor, a transmembrane tyrosine kinase encoded by the MET proto-oncogene (c-MET). The signaling cascade triggered by HGF begins with phosphorylation of the receptor and is mediated by concomitant activation of different cytoplasmic effectors that bind to the same multifunctional binding site. The c-MET mediated response includes two key pathways involved in cell survival and mitogenesis [8]. The first; protein kinase B (PKB/Akt) is activated by phosphoinositide 3-kinase (PI3K) and elicits cell survival [9,10]. The second; ERK1/2 (also known as p42/44 MAPK), a member of the mitogen-activated protein (MAP) kinase family, is activated by the RAS-RAF-MEK pathway and is responsible for mitogenesis [11]. A third response of HGF is the branching morphogenesis which next to the PKB and ERK pathways requires involvement of the signal transducer and activator of transcription (STAT) 3 pathway [12].

It is well established that an increase of TGF-β1 in liver promotes the formation of extracellular matrix (ECM) components and suppresses hepatocyte proliferation [13,14]. Prolonged overexpression of TGF-β1 in non-parenchymal cells causes hepatic fibrosis in humans and experimental animals. In several fibrosis models, fibrotic lesions are associated with an increase in collagens and TGF-β1 mRNAs [15]. The intracellular pathway that is activated by TGF-β1 receptors is mediated by Smads. Smad2 is activated via carboxy-terminal phosphorylation by TGF-β1 type I receptor kinases. When bound with co-Smads, they act as TGF-β1-induced transcriptional activators of target genes [16].

Cell homeostasis is the result of balance between cell death, cell proliferation, and growth-arrest. Therefore we investigated expression levels of pro-apoptotic Fas ligand and caspase-3, anti-apoptotic Bcl-2 [17], cell-cycle stimulating TGFα, and cell-cycle inhibitor p27kip. All of these gene-products are regulated directly or indirectly by PKB [9].

The present study was designed to describe the differential gene-expression of the above indicated crucial pathways involved in growth/regeneration, fibrosis, and cellular homeostasis in liver tissues of dogs with CPSS (reduced growth/regeneration without fibrosis) and PPVH (reduced growth/regeneration and fibrosis) in comparison with healthy animals. These simple congenital dog models may be used to unravel the roles of different gene products in those pathways. These well-defined large animal models are intended to serve as the first spontaneous liver diseases to investigate novel regenerative/anti-fibrotic therapies, such as HGF treatment. This study may also serve as a basis for future comparison with more complex diseases like chronic hepatitis and cirrhosis.

## Results

### Histological grading of fibrosis

**Figure 1:**
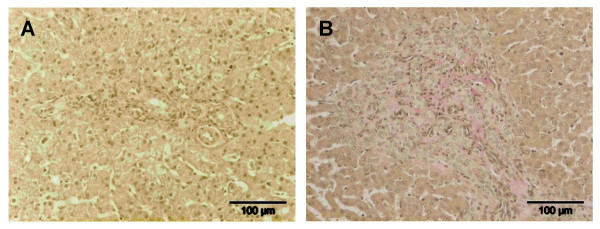
**Histological grading of fibrosis**. (A) CPSS, Portal area without recognizable portal vein and arteriolar proliferation. Van Gieson stain. (B) PPVH, Markedly enlarged portal area with fibrosis and extensive arteriolar and ductular proliferation. Van Gieson stain.

No fibrosis was seen in liver biopsies of CPSS dogs. In the PPVH dogs histological examination revealed slight portal fibrosis in one dog, slight to moderate portal fibrosis associated with slight to moderate centrolobular fibrosis in four dogs, and marked portal fibrosis with biliary proliferation in three dogs. The control dogs showed a normal liver without fibrosis. Examples of histological examination of CPSS and PPVH are included as Figures 1A and 1B, respectively.

### HGF/c-MET signaling pathway involved in regeneration and growth

**Figure 2:**
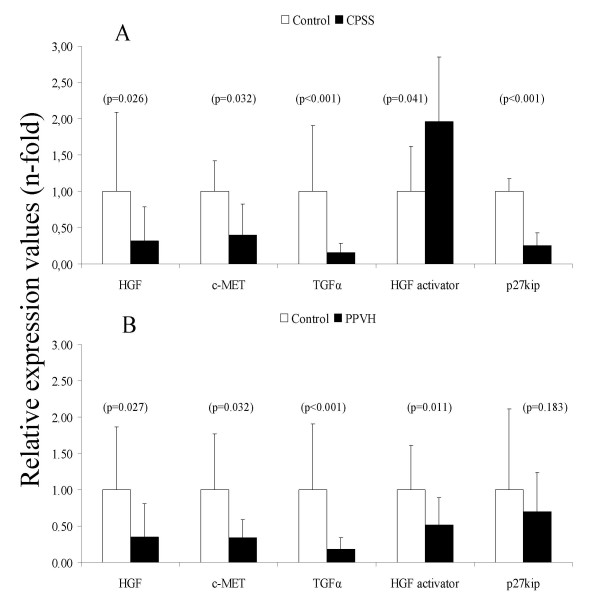
**Quantitative real-time PCR of genes involved in regeneration and growth**. Representative data of mRNA levels of congenital portosystemic shunt (CPSS, n = 11 dogs) is shown in (A). Representative data of mRNA levels of primary portal vein hypoplasia (PPVH, n = 8 dogs) is shown in (B). Data represent mean ± 2SD.

One of the main *in vivo *events during regeneration and growth is the signaling via phosphorylation of the HGF receptor c-MET. Q-PCR analysis revealed that HGF mRNA levels in both CPSS and PPVH were decreased three-fold in comparison with healthy dogs (Figure 2). Moreover, the c-MET levels in CPSS and PPVH were significantly decreased (two- and three-fold, respectively). The levels of the mRNAs for TGFα (proliferation) were decreased six-fold in both CPSS and PPVH. The serine-protease HGF activator mRNA was doubled in dogs with CPSS. In contrast, it was halved in dogs with PPVH. Although not significantly in dogs with CPSS, the cell-cycle inhibitor p27kip mRNA was decreased in both conditions.

### TGF-β1 cascade signaling pathway involved in fibrosis

**Figure 3:**
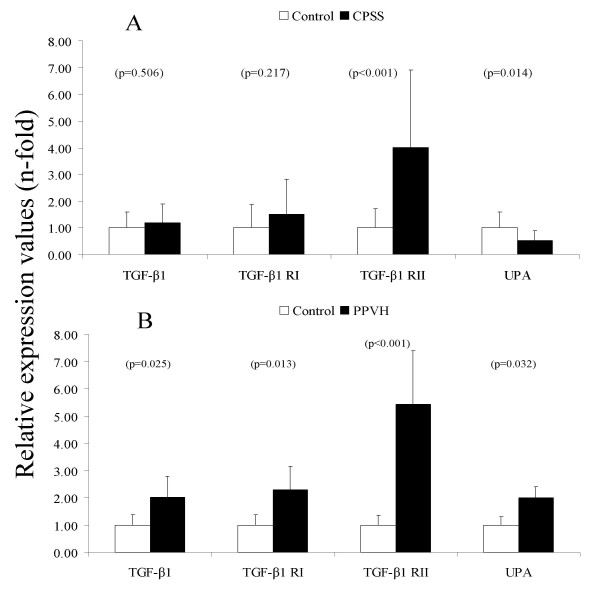
**Quantitative real-time PCR of genes involved in fibrosis**. Representative data of mRNA levels of congenital portosystemic shunt (CPSS, n = 11 dogs) is shown in (A). Representative data of mRNA levels of primary portal vein hypoplasia (PPVH, n = 8 dogs) is shown in (B). Data represent mean ± 2SD.

The fibrosis signaling pathway is activated through bindings of the active TGF-β1 dimer to the heteromeric type-I and type-II serine/threonine receptor kinases. As shown in Figure 3, TGF-β1 mRNA levels were increased two-fold in dogs with PPVH, whereas the levels in dogs with CPSS were not changed significantly. The receptor type-I, was induced in both liver diseases but only significantly in PPVH. Receptor type-II was increased in both CPSS and PPVH (4- and 5-fold, respectively), indicating an increased binding capacity. One of the proteolytic enzymes involved in activation of TGF-β1 is urokinase plasminogen activator (uPA). The uPA mRNA level was decreased two-fold in dogs with CPSS and, in contrast, doubled in dogs with PPVH.

### Gene-expression of apoptosis-related signaling proteins and hypoxia induced factor

**Figure 4:**
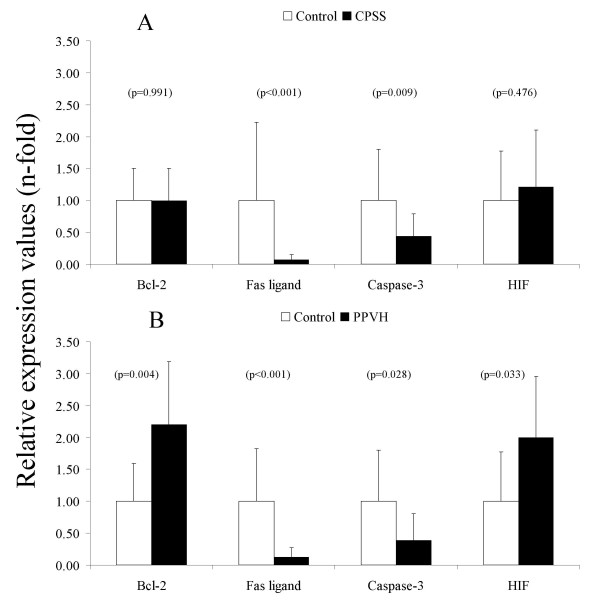
**Quantitative real-time PCR of apoptosis genes and a hypoxia related gene**. Representative data of mRNA levels of congenital portosystemic shunt (CPSS, n = 11 dogs) is shown in (A). Representative data of mRNA levels of primary portal vein hypoplasia (PPVH, n = 8 dogs) is shown in (B). Data represent mean ± 2SD.

We measured three well-known basic apoptotic components of which two are pro-apoptotic (caspase-3 and Fas ligand) and one is anti-apoptotic (Bcl-2). Figure 4 shows that pro-apoptotic mediator Fas ligand was severely inhibited in both dogs with CPSS and in dogs with PPVH (14- and 8-fold, respectively). Moreover, caspase-3 was halved in both CPSS and PPVH. On the other hand, no induction of the anti-apoptotic Bcl-2 was seen in dogs with CPSS, whereas Bcl-2 in dogs with PPVH was doubled. The mechanisms underlying progressive fibrosis are unknown, but fibrosis and hypoxia could have been a fibrogenic stimulus. Hypoxia coordinately up-regulates matrix production and hypoxia induced factor 1 alpha (HIF1α) [18]. These direct hypoxic effects on the expression of genes involved in fibrogenesis was shown in our dogs with PPVH which indeed had elevated levels of HIF1α.

### Gene-expression of extracellular matrix gene products

**Figure 5:**
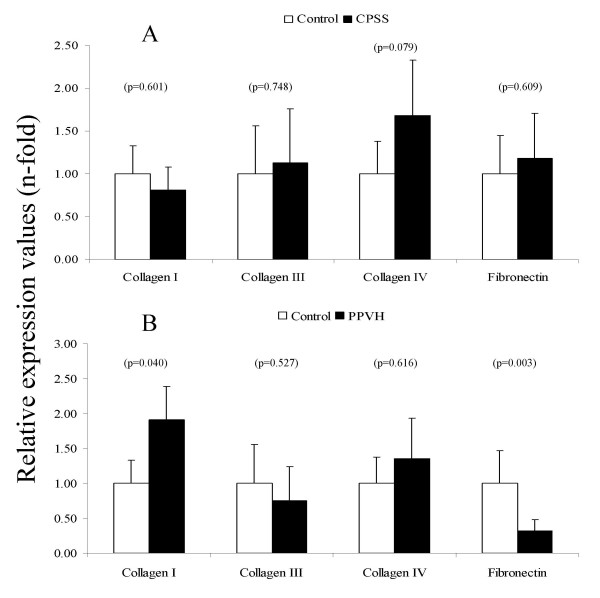
**Quantitative real-time PCR of extracellular matrix gene products**. Representative data of mRNA levels of congenital portosystemic shunt (CPSS, n = 11 dogs) is shown in (A). Representative data of mRNA levels of primary portal vein hypoplasia (PPVH, n = 8 dogs) is shown in (B). Data represent mean ± 2SD.

The analysis of ECM expression was performed on three collagens (I, III and IV) and one glycoprotein (fibronectin). Interstitial collagens types I and III are the most commonly found collagens, collagen type IV is a basal membrane collagen. In Figure 5, collagen I was shown to be significantly increased in PPVH (two-fold), whereas CPSS was unchanged. Collagen III and IV were not significantly changed in both groups. Fibronectin showed to be halved in the CPSS group where PPVH remained normal.

### Western blot analysis of HGF, c-MET, PKB, STAT3, ERK, TGF-β1, Smad2, Collagen I, and Caspase-3

**Figure 6:**
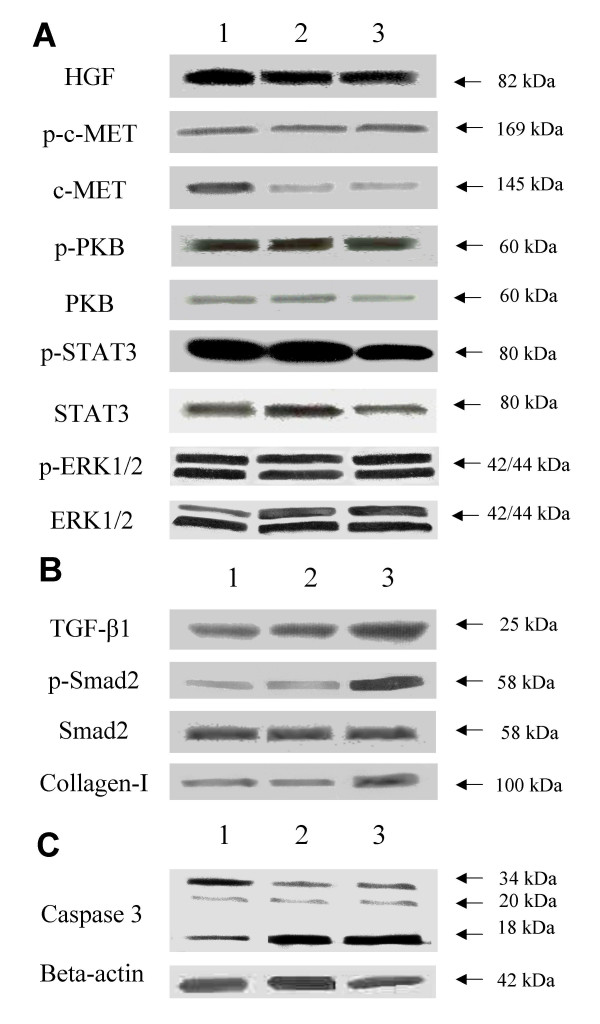
**Western blot analysis of liver homogenates of controls, CPSS, and PPVH**. Detection of HGF, c-MET, PKB, STAT, and ERK shown in (A), detection of the TGF-β1, Smad2, and Collagen I in (B), and detection of the Caspase-3protein, uncleaved/inactive 34 kDa, and cleaved/active products of 20 kDa and 13 kDa in (C). Western blot analysis of liver homogenates (n = 6 dogs per group, randomly chosen from original group). Lane samples: 1 = control; 2 = congenital portosystemic shunt; 3 = primary portal vein hypoplasia.

PKB plays a pivotal role in liver regeneration and growth upon activation of the c-MET-HGF signaling pathway [10]. Western blot analysis of HGF showed an immunoreactive band at 82 kDa with no apparent quantitative differences (Figure 6A). Non-phosphorylated c-MET was detected in all samples, where it was present as an immunoreactive band of 145 kDa. Results showed a decrease in the amount of c-MET in both diseases. On the other hand, the anti-phosphorylated c-MET antibody showed an immunoreactive band in all samples with no apparent quantitative differences. Non-phosphorylated PKB was detected in all samples, where it was present as a single band of 60 kDa. The anti-phosphorylated PKB antibody showed an immunoreactive band in all samples. Two immunoreactive bands at 42 and 44 kDa representing the MAP kinase ERK1/2 showed to be equally present at the protein level between the diseased groups and healthy controls. Interestingly, this also applied for the phosphorylated form where no apparent quantitative differences were found. The 80 kDa STAT3 protein showed a similar result with no apparent quantitative differences in the non-phosphorylated form; however, the STAT3 protein seemed to be somewhat less phosphorylated at the serine 727 residue in the PPVH group. TGF-β1 exerts its actions through complex intracellular signaling pathways. All downstream signaling routes following binding of an active TGF-β1 to its receptors type-I and II elicit phosphorylation of Smad2. TGF-β1 was seen in all diseases as a single band of 25 kDa under non-denaturing conditions (Figure 6B). Interestingly, the amount of TGF-β1 was induced in PPVH compared to CPSS and controls. Non-phosphorylated Smad2 was detected in all samples, where it was present as a single band of 58 kDa, with no apparent changes in quantity. Also interestingly, the anti-phosphorylated Smad2 antibody showed a slight band in CPSS whereas in PPVH a phosphorylated Smad2 is clearly present. Moreover, anti-collagen I showed an increase in the amount of protein in PPVH compared to CPSS and healthy controls, all together emphasizing the differences in fibrosis between CPSS and PPVH. Although reduced in the CPSS and PPVH group, inactive or uncleaved caspase-3 was detected in all samples (Figure 6C), where it was present as a single band of 34 kDa. Finally, the processed forms of 20 and 13 kDa showed to be increased in CPSS and PPVH towards healthy controls.

## Discussion

In order to analyze the possibility of growth factor therapy, two congenital canine liver diseases were molecularly dissected. The expression of a total of 17 gene products involved in liver growth/regeneration, fibrosis, ECM, and cellular homeostasis was measured and normalized to the average amount of two reference genes (Q-PCR). Western blot analysis confirmed the quantitative mRNA results and, furthermore, showed activated pathways. These two independent techniques provided insight into the effects of portal venous hypoperfusion in two canine hepatic diseases; congenital portosystemic shunt (CPSS) without fibrosis and primary portal vein hypoplasia (PPVH) with fibrosis. Taken together, the obtained data provided insights in the feasibility for HGF-treatment.

The normalization performed in this study was obtained by averaging the amount of two different reference genes, glyceraldehyde-3-phosphate dehydrogenase (GAPDH) and hypoxanthine phosphoribosyl transferase (HPRT). No samples were more than 5 percent apart from the individual measured reference genes levels (data not shown). This normalization strategy, using the average amount of two reference genes, is taken as a prerequisite for accurate Q-PCR expression profiling which enables us to measure small expression differences and allows the study of their biological relevance [19].

It is well known that HGF plays an essential role in development [20] and regeneration of the liver, and increases hepatocyte viability. The found decrease in gene-expression of both HGF and its receptor agrees with the reduced liver size in these canine disorders. However, and in contrast to the c-MET levels which correlate nicely with the found protein levels, the amount of HGF mRNA does not seem to reflect protein levels. This can be contributed to HGF which can be a paracrine but also an endocrine factor. Extra-hepatic HGF could have been present in the pancreas or intestinal tract [21].

Although HGF and c-MET mRNA levels were decreased, downstream targets of this tyrosine cascade signaling pathway were still active. Downstream targets, such as Fas ligand and p27kip, were chosen as direct or indirect targets of the HGF-cMET-PI3K-PKB axis. Fas ligand transcription is regulated by FOXO (forkhead box, sub-group "O" transcription factors). Therefore, the decrease in Fas ligand can be explained by an active PKB which directly phosphorylates FOXO [22]. A similar result can be seen in the reduced levels of p27kip mRNA, as this is down-regulated at the gene-transcription level by active PKB [23]. Combined, this indicates that PKB is active in both diseases, which was confirmed by Western blot analysis. It remains to be seen whether other receptor tyrosine kinases (e.g., EGF receptor or insulin receptor) activate this pathway in these dogs [24]. Next to the activated PKB pathway, we have analyzed other c-MET mediated responses in CPSS and PPVH. ERK1/2 showed to be activated in both diseases to a similar level as the healthy controls. The significance of the slightly reduced phosphorylated STAT3 in PPVH, which is phosphorylated by HGF on serine 727 [25], needs to be further investigated. Taken together, the pathways which elicit all major biological functions of c-MET showed to be active in CPSS and PPVH.

Prolonged or overexpression of TGF-β1 acts to suppress cell proliferation, and induces a deposition of ECM proteins, resulting in fibrosis in major organs such as liver [26,27]. We showed that in PPVH the TGF-β1 pathway through Smad2 is activated, consistent with the fibrosis seen in PPVH. Measurements on fibrosis related gene products revealed no elevated activity of the TGF-β1 pathway in CPSS. Gene expression levels related to the TGF-β1 pathway, including its receptors, and the proteolytic activator of TGF-β1 (uPA) were elevated in PPVH, thus indicating an active Smad pathway that could subsequently lead to fibrosis. Western blot analysis confirmed found TGF-β1 levels. Measurements on collagen gene-expression, especially collagen I, confirm the current paradigm of TGF-β1 signaling in fibrous tissues like PPVH [28]. Contrary, non-fibrotic CPSS did not show any alterations in collagen expression. The observation of phosphorylated Smad2 in healthy liver tissue showed that the phosphorylation of Smad2 is a dynamic process and has already been described in other publications [29,30].

The expressions of the pro-apoptotic genes Fas ligand and caspase-3 were clearly decreased. Bcl-2 gene-expression was elevated two times in PPVH; but not in CPSS (Figure 4). Western blot analysis showed that the unprocessed form of caspase-3 was present in lesser amount in CPSS and PPVH; however, the amount of processed or active bands compared to healthy control was higher in the diseases compared to healthy controls. This indicates that although the total amount of caspase-3 is lower, there is more cleavage of the caspase-3 to its active forms in the diseases, possibly leading to an increase in apoptosis.

Both HGF and TGF-β1 need extracellular processing to become biologically active. The serine protease HGF activator is responsible for activation of proHGF [31]. Our studies revealed that HGF activator gene-expression was doubled in dogs with CPSS and halved in case of PPVH. This indicated an increased HGF activation in CPSS. Although levels of HGF activator were reduced in PPVH, this does not necessarily indicate a lack of extracellular processing of HGF. Interestingly uPA, the activator of TGF-β1, was expressed at an increased level in dogs with PPVH. This may, via active TGF-β1-receptor interaction, indicate an activation of Smads and thus the formation of collagens.

Differential gene expression measurements on hepatic diseases have been performed in the past; nevertheless, little is known about levels of genes that play an important role in fibrosis. There have been measurements on cirrhosis in man and rat that indicate an up or down-regulated expression of several proteins [32]. Although these results might be significant in severe forms of fibrosis, these data depict an end-point of the disease whereas earlier stages may be more informative.

Regeneration with recombinant HGF has been achieved in rodent models of liver failure [33,34]. Moreover, besides its regenerative capacity, HGF is known to have an antifibrogenic effect [35,36] and thus reduces or prevent fibrosis in PPVH. TGF-β1 intervention to halt the progression of liver fibrosis and positively effect regeneration, has been applied successfully [37] even in cirrhosis [38]. The measured gene products involved in fibrosis in PPVH make it a good spontaneous animal model to investigate new therapeutic strategies to influence the HGF and/or TGF-β1 pathways *in vivo*. Furthermore, most fibrogenic models are induced by toxins such as dimethylnitrosamine (DMN), CCl_4_, or thioacetamide [39]. The canine PPVH model is not drug-induced; therefore, may be better to compare with human diseases and thus fill the gap between induced rodent models and human diseases.

This study is the first to measure expression profiles of crucial pathways of liver growth/regeneration, fibrosis, and hepatic homeostasis in spontaneous canine liver diseases. The present findings in two diseases with relatively simple pathogenesis may also serve as basis for evaluation of more complex diseases like hepatitis and cirrhosis. Evaluation of such complex diseases in dogs is highly suitable for comparative studies on the roles of different pathways in the pathogenesis of liver diseases in man. Two further conclusions can be deduced from the data presented here. First, the pathophysiological differences between CPSS and PPVH can nicely be explained by the Q-PCR measurements and Western blots. Second, although c-MET levels were reduced, downstream signaling seemed to be functional and provides a rational background to design controlled studies for HGF-supplementation in CPSS and PPVH.

## Methods

### Animals

All samples are obtained from different dog breeds appearing in the clinic with spontaneous diseases. Samples were randomly chosen and aimed to encompass different dog-breeds and both sexes in each group. The procedures were approved by the Ethical Committee as required under Dutch legislation.

### Groups

The congenital portosystemic shunt (n = 11 dogs) and primary portal vein hypoplasia group (n = 8 dogs) were compared with a group of healthy dogs (n = 11 dogs). The inclusion criteria for CPSS were increased fasting plasma ammonia concentration, abnormal ammonia tolerance test (peak ammonia ≥ 150 μmol/l plasma) and ultrasonographic visualization of a small liver and a congenital portosystemic shunt with a diameter as wide as the portal vein trunk. The presence of the shunt was further confirmed with surgery, during which a wedge liver biopsy was taken and immediately put in liquid nitrogen and stored at -70°C, until analysis. In CPSS there is no portal hypertension. The inclusion criteria for PPVH were the visualization of a small liver with ultrasonography, presence of multiple small acquired portosystemic collaterals due to portal hypertension, and an abnormal ammonia tolerance test (peak ammonia ≥ 150 μmol/l plasma). Liver tissue of dogs with PPVH was obtained under local anaesthesia by ultrasound-guided biopsy with a true cut 16G biopsy needle. Two biopsies were immediately immersed in liquid nitrogen, and stored at -70°C, until analysis. The healthy control dogs were age-matched, and had AP, ALT, and fasting bile acids in plasma within the reference range. Ultrasonographically the control dog livers had a normal size, shape, and structure, and there were no histological abnormalities in stained histological sections.

### Histological grading of fibrosis

Liver samples were fixed in 10% buffered formalin and routinely embedded in paraffin. Sections (4 μm) were stained with haematoxylin-eosin, the Van Gieson stain, and the reticulin stain according to Gordon and Sweet. Histologically, the presence of fibrosis was evaluated semi-quantitatively (absent, slight, moderate, or marked) as well as with respect to its localization. Fibrosis scoring was performed according to Scheuer, a defined scoring method for fibrosis in hepatitis. The slides were independently examined by one certified veterinary pathologist.

### RNA isolation and reverse-transcription polymerase chain reaction

Total cellular RNA was isolated from each frozen canine liver tissue in duplicate, using the RNeasy Mini Kit (Qiagen, Leusden, The Netherlands) according to the manufacturer's instructions. The RNA samples were treated with Dnase-I (Qiagen Rnase-free DNase kit). In total 3 μg of RNA was incubated with poly(dT) primers at 42°C for 45 min, in a 60 μl reaction volume, using the Reverse Transcription System from Promega (Promega Benelux, Leiden, The Netherlands).

### Quantitative measurements of the mRNA levels of HGF, TGF-β1, and other related signaling molecules

Q-PCR based on the high affinity double-stranded DNA-binding dye SYBR^® ^green I (BMA, Rockland, ME) was performed in triplicate in a spectrofluorimetric thermal iCycler^® ^(BioRad, Veenendaal, The Netherlands). Data were collected and analyzed with the provided application software. For each Q-PCR, 2 μl (of the 2 times diluted stock) of cDNA was used in a reaction volume of 50 μl containing 1× manufacturer's buffer, 2 mM MgCl_2_, 0.5 × SYBR^® ^green I, 200 μM dNTP's, 20 pmol of both primers, 1.25 units of AmpliTaq Gold (Applied Biosystems, Nieuwerkerk a/d IJssel, The Netherlands), on 96-well iCycler iQ plates (BioRad). Primer pairs, depicted in Table 1, were designed using PrimerSelect software (DNASTAR Inc., Madison, WI). All PCR protocols included a 5-minute polymerase activation step and continued for 40 cycles at 95°C denaturation for 20 sec, annealing for 30 sec and elongation at 72°C for 30 sec with a final extension for 5 min at 72°C. Annealing temperatures were optimized at various levels ranging from 56°C till 67°C (Table 1). Melt curves (iCycler, BioRad), agarose gel electrophoresis, and standard sequencing procedures were used to examine each sample for purity and specificity (ABI PRISM 3100 Genetic Analyser, Applied Biosystems). Standard curves constructed by plotting the relative starting amount versus threshold cycles were generated using serial 4-fold dilutions of pooled cDNA fractions from both healthy and diseased liver tissues. The amplification efficiency, E (%) = (10^(1/-s)^-1) * 100 (s = slope), of each standard curve was determined and appeared to be > 95%, and < 105%, over a wide dynamic range. For each experimental sample, the amount of the gene of interest, and of the endogenous references glyceraldehyde-3-phosphate dehydrogenase (GAPDH) and hypoxanthine phosphoribosyl transferase (HPRT) were determined from the appropriate standard curve in autonomous experiments. If relative amounts of GAPDH and HPRT were constant for a sample, data were considered valid and the average amount was included in the study (data not shown). Results were normalized according to the average amount of the endogenous references. The normalized values were divided by the normalized values of the calibrator (healthy group) to generate relative expression levels [40].

**Table 1:** Nucleotide Sequences of Dog-Specific Primers for Real-Time Q-PCR.

**Gene**	**Primer**	**Sequence (5'-3')**	**°C**	**Product size (bp)**	**Accession number**
GAPDH	Forward	TGT CCC CAC CCC CAA TGT ATC	58	100	AB038240
	Reversed	CTC CGA TGC CTG CTT CAC TAC CTT			
HPRT	Forward	AGC TTG CTG GTG AAA AGG AC	56	100	L77488/L77489
	Reversed	TTA TAG TCA AGG GCA TAT CC			
HGF	Forward	AAA GGA GAT GAG AAA CGC AAA CAG	58	92	BD105535
	Reversed	GGC CTA GCA AGC TTC AGT AAT ACC			
c-MET	Forward	TGT GCT GTG AAA TCC CTG AAT AGA AATC	59	112	AB118945
	Reversed	CCA AGA GTG AGA GTA CGT TTG GAT GAC			
TGFα	Forward	CCG CCT TGG TGG TGG TCT CC	63	136	AY458143
	Reversed	AGG GCG CTG GGC TTC TCG T			
HGF activator	Forward	ACA CAG ACG TTT GGC ATC GAG AAG TAT	60	128	AY458142
	Reversed	AAA CTG GAG CGG ATG GCA CAG			
p27kip	Forward	CGG AGG GAC GCC AAA CAG G	60	90	AY455798
	Reversed	GTC CCG GGT CAA CTC TTC GTG			
TGF-β1	Forward	CAA GGA TCT GGG CTG GAA GTG GA	66	113	L34956
	Reversed	CCA GGA CCT TGC TGT ACT GCG TGT			
TGF-β1 R I	Forward	CAG TCA CCG AGA CCA CAG ACA AAG T	59	101	AY455799
	Reversed	TGA AGA TGG TGC ACA AAC AAA TGG			
TGF-β1 R II	Forward	GAC CTG CTG CCT GTG TGA CTT TG	61	116	AY455800
	Reversed	GGA CTT CGG GAG CCA TGT ATC TTG			
UPA	Forward	CTG GGG AGA TGA AGT TTG AGG TGG	64.5	105	AY455801
	Reversed	TGG AAC GGA TCT TCA GCA AGG C			
Bcl-2	Forward	TGG AGA GCG TCA ACC GGG AGA TGT	61	87	AB116145
	Reversed	AGG TGT GCA GAT GCC GGT TCA GGT			
Fas Ligand	Forward	GGG GTC AGT CCT GCA ACA ACA A	54	94	AY603042
	Reversed	ATC TTC CCC TCC ATC AGC ATC AG			
Caspase-3	Forward	ATC ACT GAA GAT GGA TGG GTT GGT	58	140	AB085580
	Reversed	GAA AGG AGC ATG TTC TGA AGT AGC ACT			
HIF1α	Forward	TTA CGT TCC TTC GAT CAG TTG TCA	61	106	AY455802
	Reversed	GAG GAG GTT CTT GCA TTG GAG TC			
Collagen I	Forward	GTG TGT ACA GAA CGG CCT CA	61	111	AF056303
	Reversed	TCG CAA ATC ACG TCA TCG			
Collagen III	Forward	ATA GAG GCT TTG ATG GAC GAA	65	134	AB042266
	Reversed	CCT CGC TCA CCA GGA GC			
Collagen IV	Forward	CAC AGC CAG ACA ACA GAT GC	67	151	U07888
	Reversed	GCA TGG TAC TGA AGC GAC G			
Fibronectin	Forward	AGG TTG TTA CCA TGG GCA	61	91	U52106
	Reversed	GCA TAA TGG GAA ACC GTG TAG			

### Statistical analysis

A Kolmogorov-Smirnov test was performed to establish a normal distribution and a Levene's test for the homogeneity of variances. All samples included in this study were normally distributed and homogeneous in variance. The statistical significance of differences between diseased and control animals was determined by using the Student's *t*-test. A p-value < 0.05 was considered statistically significant. Analysis was performed using SPSS software (SPSS Benelux BV, Gorinchem, The Netherlands).

### Western blot analysis

Used antibodies are described in Table 2. For Western blot analysis 30 mg of liver tissue from at least six samples of each group (n = 6 dogs per group, randomly chosen from original group) were pooled and analyzed. Liver tissues were homogenized in RIPA buffer containing 1% Igepal, 0.6 mM phenylmethylsulfonyl-fluoride, 17 μg/ml aprotinine, and 1 mM sodium-orthovanadate (Sigma chemical Co., Zwijndrecht, The Netherlands). Protein concentrations were obtained using a Lowry-based assay (DC Protein Assay, BioRad). Twenty μg of protein of the supernatant was denatured for 3 min at 95°C and electroforesed on 7.5% Tris-HCl polyacrylamide gels (BioRad) and the proteins were transferred onto Hybond-C Extra Nitrocellulose membranes (Amersham Biosciences Europe, Roosendaal, The Netherlands) using a Mini Trans-Blot^® ^Cell blot-apparatus (BioRad). Immunodetection was based on an ECL Western blot analysis system, performed according to the manufacturer's instructions (Amersham Biosciences Europe). The membranes were incubated with 4% ECL blocking solution in TBS for 1 hour under gentle shaking. The incubation of the primary antibody was performed at 4°C over-night for all antibodies (see Table 2) in TBS with 0.1% Tween-20 (Boom B.V., Meppel, The Netherlands). After washing, the membranes were incubated with their respective horseradish peroxidase-conjugated secondary antibody (R&D systems, Europe Ltd., Abingdon, UK) at room temperature for 1 h and exposed to Kodak BioMax Light-1 films (Sigma chemical Co.). Densitometric analysis of immunoreactive bands was performed with a Gel Doc 2000 system coupled to the Quantity One 4.3.0 Software (BioRad).

**Table 2:** Used antibodies in Western blot experiments.

**Antigen**	**Product Size (kDa)**	**Dilution**	**Manufacturer**	**Secondary antibody**	**Dilution**
HGF	82	1:100	Neomarkers	Anti-mouse HRP	1:20,000
p-c-MET (Tyr 1230/1234/1235)	145	1:750	Abcam	Anti-rabbit HRP	1:20,000
c-MET	145	1:750	Sigma	Anti-goat HRP	1:20,000
p-PKB (Thr 308)	60	1:1,000	Cell-Signaling	Anti-mouse HRP	1:20,000
PKB	60	1:250	BD Biosciences	Anti-mouse HRP	1:20,000
p-STAT3 (Ser 727)	86	1:1,000	Cell Signalling	Anti-rabbit HRP	1:20,000
STAT3	86	1:2,500	BD Biosciences	Anti-rabbit HRP	1:20,000
p-Erk1/2 (Thr 202/Tyr 204)	42/44	1:1,500	Cell Signalling	Anti-rabbit HRP	1:20,000
ERK1/2	42/44	1:1,000	Cell Signalling	Anti-rabbit HRP	1:20,000
TGF-β1	25	1:1,000	Abcam	Anti-rabbit HRP	1:20,000
p-Smad2 (Ser 465/467)	58	1:2,000	Cell-Signaling	Anti-rabbit HRP	1:20,000
Smad2	58	1:500	BD Biosciences	Anti-mouse HRP	1:20,000
Collagen I	95/210	1:500	Calbiochem	Anti-mouse HRP	1:20,000
Caspase-3	34/20/18	1:1,000	Calbiochem	Anti-rabbit HRP	1:20,000
Beta-actin (pan Ab-5)	42	1:2,000	Neomarkers	Anti-mouse HRP	1:20,000

## Competing interests

The author(s) declare that they have no competing interests.

## Authors' contributions

BS performed most Q-PCR measurements and wrote the manuscript. LP participated in the setup of Q-PCR measurements and helped to draft the manuscript. TI histochemically examined samples described in this manuscript. BA helped perform the0 Western blot experiments. JIJ histochemically examined samples described in this manuscript. FS helped collect all samples. JR participated in the study design and helped to draft the initial manuscript. All authors read and approved the final manuscript.
